# Impact of gender mismatch on corneal graft rejection and rejection-related graft failure in repeat penetrating keratoplasty

**DOI:** 10.1371/journal.pone.0276043

**Published:** 2022-10-31

**Authors:** Varintorn Chuckpaiwong, Passara Jongkhajornpong, Pongthep Rajsirisongsri, Vachira Sontichai, Sarayut Nijvipakul, Kaevalin Lekhanont

**Affiliations:** 1 Faculty of Medicine, Department of Ophthalmology, Ramathibodi Hospital, Mahidol University, Bangkok, Thailand; 2 Faculty of Medicine, Chakri Naruebodindra Medical Institute, Ramathibodi Hospital, Mahidol University, Samut Prakan, Thailand; Saarland University, GERMANY

## Abstract

**Purpose:**

To explore the impact of gender mismatch on corneal allograft rejection and rejection-related graft failure in patients with repeat penetrating keratoplasty (PK).

**Methods:**

A retrospective cohort was conducted at Ramathibodi Hospital, Bangkok, Thailand. Patients with repeat PK and follow-up period of at least 6 months were recruited. Demographic data, pre-operative ocular findings and donor information were collected. Patients were divided into 2 groups according to gender mismatch between donor and recipient (male to female vs others). Survival analysis was performed using the Kaplan-Meier method. The association between risk factors and the outcomes of graft rejection and rejection-related graft failure were analyzed using Cox proportional hazards regression.

**Results:**

Of 68 patients with repeat PK, 26 patients (38.2%) were gender mismatched. There was no difference in 3-year rejection-free survival and graft survival between patients with gender mismatch and others (p = 0.698 and p = 0.402, respectively). Younger recipients (< 40 years) showed a significantly higher rejection rate compared to older recipients (hazard ratio; HR = 3.14, 95% confidence interval; CI 1.15 to 8.58), while patients with multiple PK (> 2 times) were found to be significantly associated with higher rejection-related graft failure compared to patients with 2 times or less PK (HR = 2.72, 95% CI 1.03 to 7.21). After multivariate analysis, only younger recipients demonstrated a statistical significance on graft rejection (adjusted HR = 2.86, 95% CI 1.04 to 7.84).

**Conclusions:**

Gender mismatch might not impact corneal graft rejection or rejection-related graft failure in patients with repeat PK. Younger age was found to be a strong factor associated with graft rejection. Gender matching might not be compulsory for repeat PK.

## Introduction

Corneal transplantation is the most common and successful procedure among all tissue and organ transplantation [1]. The main mechanisms of immune privilege in normal cornea include the absence of blood and lymphatic vessels, low expression of major histocompatibility complex (MHC) and the ability to induce anterior chamber-associated immune deviation (ACAID), which all contribute to the lower rate of tissue rejection compared to solid organ transplantation [2]. However, this protective system becomes compromised in eyes with history of previous corneal transplantation because of presensitization, immune memory, inflamed and vascular surroundings from previous surgeries [3]. Graft failure was reported as one of the leading indications of penetrating keratoplasty (PK) in several countries, ranging from 17% - 27% [4–7]. This substantial proportion of eyes with repeat PK are inevitably prone to develop corneal allograft rejection, which potentially leads to irreversible graft failure. Data from The Collaborative Corneal Transplantation Studies (CCTS) showed that the rate of rejection-related graft failure significantly increased from 8%, with no previous grafts, to approximately 40% in eyes with repeat PK [8].

Several measures for donor and recipient matching to reduce the occurrence of graft rejection in high-risk eyes have been proposed [3]. Currently, the standard guideline of tissue matching for corneal transplantation has yet to be established. Since there is no expression of the major histocompatibility complex (MHC) class I alloantigen and very low expression of MHC class II molecules on corneal graft, minor histocompatibility antigens (mHAs) may possibly play a dominant role than MHC on corneal allograft rejection [9,10]. A few studies in animals demonstrated that MHC class II molecule-specific CD4+ T cells played a major part in acute and subacute corneal graft rejection [11–13], however, a recent clinical trial in humans has failed to show the effect of HLA class II matching on corneal graft rejection [14]. H-Y antigens are a group of minor mHAs encoded on the Y chromosome and expressed on various cell types and tissues including cornea, therefore, they potentially serve as important immunogenic targets in gender-mismatched transplantation [15]. H-Y mismatched (male to female) organ transplantation transfers these immune targets from male donor to female recipient and consequently contributes to allograft rejection. A large study based on big data from National Health Service Blood and Transplant in UK demonstrated that H-Y mismatched corneas had greater risk of graft rejection or failure compared to matched corneas [16]. However, this finding was not consistently demonstrated in later studies from Italy and Korea [17,18]. Furthermore, the effect of gender mismatch in high-risk eyes, especially in eyes with repeat PK, has not been completely explored.

This study aimed to investigate the role of gender mismatch between corneal donors and recipients on corneal allograft rejection and rejection-related graft failure in patients with repeat PK. Our results can assist in the allocation of donor corneal grafts to recipient eyes with history of previous PK to reduce corneal graft rejection, increase corneal graft survival and improve overall clinical outcomes.

## Methods

A retrospective chart review of patients with repeat PK was conducted according to the tenets of the Declaration of Helsinki and was approved by the Ethics Committee of Ramathibodi Hospital (MURA2018/350). Consecutive patients who underwent repeat PK at our hospital from January 2010 to December 2017 and were followed up for at least 6 months were included. Patients’ characteristics including gender, age, both underlying systemic and ocular diseases, primary diagnosis, number of PK, ocular findings (peripheral anterior synechiae; PAS and deep stromal neovascularization; NV) were collected from electronic medical records. Donors’ information (i.e. endothelial cell density; ECD, time to preservation; TTP, and donor size) and source (domestic or US eye banks) were also obtained. The information of the first graft was also collected whenever available. Patients with incomplete information of both donors and recipients of repeat graft were excluded.

Experienced cornea surgeons performed PK using the same technique with 16 interrupted sutures. The standard postoperative regimen in our center included prednisolone acetate 1% every 2 hours and levofloxacin 4 times a day for 2–4 weeks, followed by prednisolone acetate 1% only, slowly tapered until 1 drop daily at 6 months after surgery. Immunosuppressive medication, apart from topical steroid, was individually prescribed according to patient health and socioeconomic status. Patients were admitted and followed-up daily until corneal epithelial defect was closed, generally within 10 days. Then, patients were evaluated at 2 weeks, 1 month, 3 months, and every 3 months up to 1 year after surgery, and then examined every 3–6 months for patient lifetime. Corneal graft rejection was defined as new onset of anterior chamber inflammation and corneal edema with or without endothelial rejection line [19]. Corneal graft failure was defined as cloudy cornea with loss of central graft clarity for a minimum of 3 consecutive months [20].

### Statistical analysis

Only one eye of each patient was analyzed and only the latest graft received by an individual patient during the study period was considered in the analysis. Mean and standard deviation (SD) or median and range; and counts with percentage were used to describe continuous and categorical data, respectively. Patients were divided into 2 groups according to gender mismatch (male donor to female recipient) and others (i.e., male donor to male recipient, female donor to male recipient, and female donor to female recipient). The associations between factors of interest (i.e., gender mismatch, recipient factors, and donor factors) and the presence of corneal graft rejection and rejection-related graft failure were analyzed using Kaplan-Meier curves with Cox proportional hazards regression. Significant factors from univariate analysis with a p-value < 0.2 were included and selected into the final model using the backward selection method. The effects of gender mismatch of the first graft on rejection and rejection-related graft failure were also evaluated in 43 patients with available information. Survival rate and hazard ratio (HR) were estimated and presented with 95% confidence interval (CI). P-values of less than 0.05 were considered statistically significant. Analyses were conducted using STATA version 16.0 (StataCorp, College Station, TX, USA).

## Results

Sixty-eight patients with repeat PK were included in the analysis. Half of the patients (35 patients, 51.5%) were female with the mean age of 63.7 (SD 15.2) years. Glaucoma was found in 46 patients (67.7%). Of these patients, most underwent PK twice (56 patients, 82.3%) and one patient underwent PK 6 times. Most corneal donors (51 patients, 75%) were from a domestic eye bank (Thai Red Cross Society) and the rest were imported from the US. Twenty-six patients (38.2%) were gender mismatched. Recipient and donor information between gender mismatch and others were compared and are presented in Table 1. No statistically significant factor was observed between both groups.

**Table 1:** Characteristics of patients with repeat penetrating keratoplasty (PK) and their donors.

Characteristics	Donor-recipient gender		P values
	Male-female (n = 26)	Others (n = 42)	
*Recipient factors*			
Age, mean (years, SD)	61.1 (18.2)	65.3 (12.9)	0.266
Number of PK (times, SD)	2.2 (0.1)	2.4 (0.9)	0.385
Underlying glaucoma	18 (69.2%)	28 (66.7%)	0.826
Peripheral anterior synechiae (PAS)	9 (34.6%)	15 (35.7%)	0.927
Corneal stromal neovascularity (NV)	10 (38.5%)	8 (19.0%)	0.078
Received immunosuppressive drugs	3 (11.5%)	4 (9.5%)	1.000
*Donor factors*			
Source Domestic US	19 (73.1%) 7 (26.9%)	33 (76.2%) 10 (23.8%)	0.773
Time to preservation (hours, SD) ≤ 12 hours > 12 hours	17 (65.4%) 9 (34.6%)	22 (50.0%) 21 (50.0%)	0.214
Endothelial cell count (cells, SD) < 2,800 cells/mm^2^ ≥ 2,800 cells/mm^2^	7 (26.9%) 19 (73.1%)	19 (45.2%) 23 (54.8%)	0.131
Graft size 7.5–8 mm < 7.5 mm or > 8 mm	17 (65.4%) 9 (34.6%)	30 (71.4%) 12 (28.6%)	0.600

Of 68 patients with repeat PK, 29 (42.6%) patients developed corneal graft rejection and 21 (30.9%) patients consequently turned to graft failure. The median follow-up time in our study was 3 years (ranged 7 months to 9 years). The overall rejection free survival rates estimated at 1, 3 and 5 years were 85.2% (95% CI 74.2%, 91.7%), 64.3% (95% CI 50.7%, 75.0%), and 47.9% (95% CI 32.8%, 61.5%), respectively. The overall rejection-related graft survival rates at 1, 3, and 5 years were 95.4% (95% CI 86.4%, 98.5%), 79.6% (95% CI 65.8%, 88.3%), and 52.3% (95% CI 34.7%, 67.3%), respectively.

### Factors associated with graft rejection

**Fig 1:**
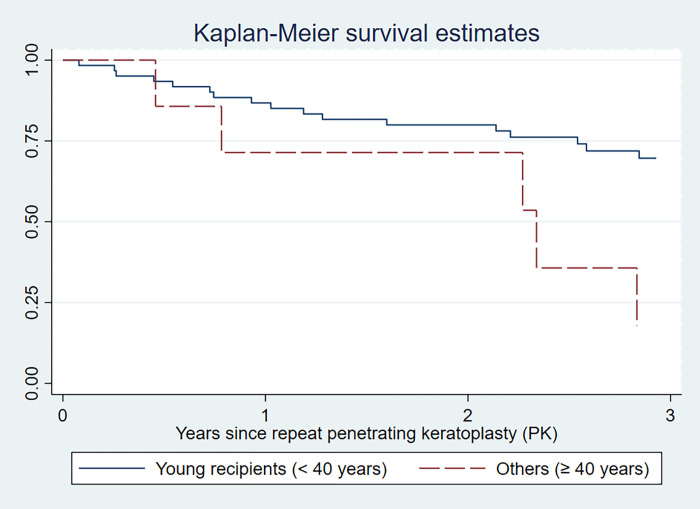
Kaplan-Meier estimate of rejection free survival for patients with repeat penetrating keratoplasty according to age of recipients.

Gender mismatch did not have a statistically significant association with graft rejection (p-value 0.698). Only young recipients (< 40 years) had a significantly increased risk of graft rejection. The Kaplan-Meier rejection-free survival estimated at 3 years was lower in young patients (17.9%; 95% CI 0.8%, 53.8%) compared to patients aged ≥ 40 years (69.7%; 95% CI 55.6%, 80.1%) as shown in Fig 1. Young recipients had a significantly increased risk for graft rejection with the HR of 3.14 (95% CI 1.15, 8.58). Other factors including glaucoma, PAS, corneal stromal NV, number of penetrating keratoplasty, donor source, ECD, time to preservation, donor size were not significantly associated with graft rejection in patients with repeat PK (Table 2).

**Table 2:** Factors associated with graft rejection in patients with repeat penetrating keratoplasty (PK).

Factors	No. of patients	No. of patients with rejection	3-year rejection free survival % (95% CI)	Univariate analysis		Multivariate analysis	
				HR (95% CI)	P-values	Adjusted HR (95% CI)	Adjusted P-values
Donor-recipient gender Male-female Others	26 42	9 (34.6%) 20 (47.6%)	67.5 (45.0, 82.4) 63.0 (45.3, 76.4)	0.85 (0.38, 1.89) 1	0.698	0.86 (0.39, 1.91)	0.712
Recipient factors							
Young age (< 40 years)	7	5 (71.4%)	17.9 (0.8, 53.8)	3.14 (1.15, 8.58)	0.025	2.86 (1.04, 7.84)	0.042
Multiple PK (> 2 times)	12	7 (58.3%)	40.0 (13.5, 65.7)	2.20 (0.93, 5.20)	0.073	2.01 (0.84, 4.80)	0.115
Underlying glaucoma	46	17 (37.0%)	68.7 (51.9, 80.6)	0.72 (0.34, 1.51)	0.391	-	-
Presence of PAS	24	13 (54.2%)	53.5 (30.2, 72.2)	1.86 (0.88, 3.89)	0.099	-	-
Presence of corneal stromal NV	18	8 (44.4%)	65.7 (38.7, 83.0)	0.99 (0.44, 2.25)	0.984	-	-
Received immunosuppressive drugs	7	3 (42.9%)	64.3 (15.1, 90.2)	1.12 (0.34, 3.71)	0.856	-	-
Donor factors							
US source	17	7 (41.2%)	56.3 (29.3, 76.4)	1.02 (0.43, 2.39)	0.965	-	-
Time to preservation > 12 hours	30	15 (50.0%)	63.1 (42.0, 78.3)	1.21 (0.58, 2.53)	0.604	-	-
ECD < 2,800 cells/mm^2^	26	11 (42.3%)	57.1 (34.4, 74.5)	0.92 (0.43, 1.96)	0.822	-	-
Extreme graft size (< 7.5 mm or > 8 mm)	21	8 (38.1%)	63.7 (38.0, 81.0)	0.68 (0.30, 1.56)	0.363	-	-

After multivariate analysis using Cox proportional hazards regression model, the effect of gender mismatch was still unchanged after adjusted with age group and number of PK. Young recipients remained the only significant factor associated with higher rate of graft rejection (adjusted HR 2.86; 95% CI 1.04, 7.84), see Table 2.

### Factors associated with rejection-related graft failure

**Fig 2:**
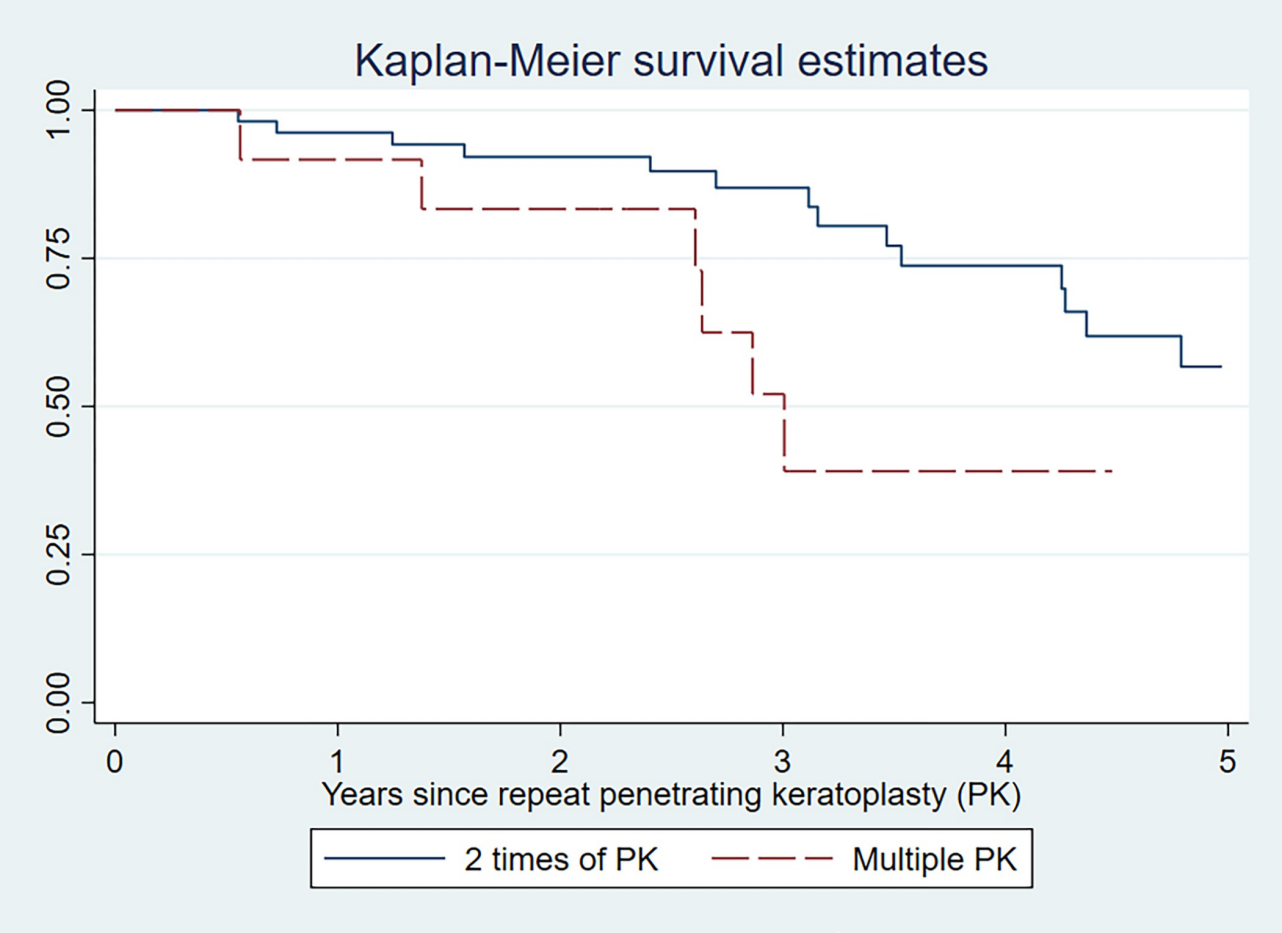
The Kaplan-Meier estimate of graft survival in patients with repeat penetrating keratoplasty according to the number of repeat keratoplasty.

Gender mismatch had no statistically significant association with rejection-related graft failure (p-value 0.402). Only number of PK was significantly associated with higher risk of rejection-related graft failure (HR 2.72; 95% CI 1.03, 7.21). The Kaplan-Meier graft survival estimated at 3 years was lower in patients with multiple PK (52.1%; 95% CI 19.8%, 76.9%) compared to patients who underwent PK twice (86.9%; 95% CI 72.8%, 94.0%) as shown in Fig 2. Young recipients showed a borderline significance for rejection-related graft failure in univariate analysis (HR 3.50, 95% CI 0.97, 12.63). Other factors were not significantly associated with rejection-related graft failure in patients with repeat PK (Table 3). After multivariate analysis, no significant factor was found to be associated with rejection-related graft failure (Table 3).

**Table 3:** Factors associated with rejection-related graft failure in patients with repeat penetrating keratoplasty (PK).

Factors	No. of patients	No. of patients with failure	3-year rejection free survival % (95% CI)	Univariate analysis		Multivariate analysis	
				HR (95% CI)	P-values	Adjusted HR (95% CI)	Adjusted P-values
Donor-recipient gender Male-female Others	26 42	10 (38.5%) 19 (45.2%)	78.4 (50.8, 91.6) 79.8 (61.9, 89.9)	0.65 (0.23, 1.79) 1	0.402	0.64 (0.23, 1.78)	0.395
Recipient factors							
Young age (< 40 years)	7	4 (57.1%)	57.1 (7.6, 88.6)	3.50 (0.97, 12.63)	0.055	2.93 (0.80, 10.70)	0.104
Multiple PK (> 2 times)	12	6 (50.0%)	52.1 (19.8, 76.9)	2.72 (1.03, 7.21)	0.044	2.62 (0.97, 7.08)	0.058
Underlying glaucoma	46	19 (41.3%)	77.5 (59.5, 88.3)	0.97 (0.40, 2.36)	0.946	-	-
Presence of PAS	24	12 (50.0%)	77.8 (50.4, 91.2)	1.98 (0.83, 4.73)	0.126	-	-
Presence of corneal stromal NV	18	7 (38.9%)	87.4 (58.1, 96.7)	0.46 (0.15, 1.44)	0.185	-	-
Received immunosuppressive drugs	7	3 (42.9%)	60.0 (12.6, 88.2)	1.43 (0.42, 4.89)	0.566	-	-
Donor factors							
US source	17	8 (47.1%)	73.0 (42.9, 89.0)	1.31 (0.50, 3.38)	0.581	-	-
Time to preservation > 12 hours	30	15 (50.0%)	79.1 (56.5, 90.8)	1.32 (0.55, 3.14)	0.532	-	-
ECD < 2,800 cells/mm^2^	26	14 (53.8%)	67.7 (43.5, 83.3)	1.42 (0.60, 3.35)	0.426	-	-
Extreme graft size (< 7.5 mm or > 8 mm)	21	8 (38.1%)	72.1 (44.7, 87.5)	0.75 (0.29, 1.94)	0.554	-	-

### Subgroup analysis of patients with available data of the first graft

There were 43 patients with available gender information of the first graft. Gender mismatch was found in 5 patients (11.6%). Of 43 patients, graft rejection and rejection-related graft failure developed in 20 patients (2 patients with gender mismatch) and 13 patients (1 patient with gender mismatch), respectively. Gender mismatch of the first graft was found to have no statistically significant association with graft rejection (HR 1.14; 95% CI 0.26–4.93, p-value 0.865) and rejection-related graft failure (HR 1.24; 95% CI 0.16–9.93, p-value 0.837) in patients with repeat PK.

## Discussion

Results of the effect of gender mismatch on corneal allograft rejection remains controversial. Our study found that gender mismatch was not significantly associated with the risk of graft rejection in patients with repeat PK. Previous evidence from Bohringer et al. showed that HLA-A1-restricted Y-antigen on chromosome Y in male donors may have a role in inducing graft rejection when they are transplanted in female recipients [21]. However, the prevalence of HLA class I varies among different ethnicities, implying that the effect of gender mismatch might differ across nations. Kim et al. conducted a study in Korea and found that gender matching did not have any influence on graft rejection and failure in patients with primary PK [18]. On the other hand, a retrospective study based on big data from NHS Blood and Transplant in the UK found that gender mismatch was a significant risk factor for graft rejection or failure in patients with Fuch’s endothelial corneal dystrophy (FECD) who underwent all types corneal transplantation [16]. However, this significant effect was not consistently observed in other corneal diseases (e.g. keratoconus and infection) or specific type of corneal transplantation (i.e. PK and deep anterior lamellar keratoplasty) [16]. Additionally, the authors did not take into account the different types of keratoplasty (i.e. PK, Descemet stripping automated endothelial keratoplasty; DSAEK, Descemet membrane endothelial keratoplasty; DMEK and deep anterior lamellar keratoplasty; DALK) and repeat transplantation. Therefore, the effect of donor-recipient gender matching on high-risk patients with repeat PK could not be over-claimed. Similar to our results, the recent prospective multicenter cohort study in Italy, considering type of transplantation and previous failed graft, observed no significant differences between gender matched and unmatched patients for both incidence of graft rejection and failures [17]. These conflicting results might be explained by the difference of HLA-A1 allele frequencies among different countries (approximately 21% in England, 10–15% in Italy, 3% in Thailand, and 2% in Korea) [22].

We found a significant increase in graft rejection rate in young patients. Recipients aged below 40 years were approximately 3 times more likely to develop graft rejection after repeat PK compared to recipients aged 40 years or older. Our finding was similar to results from previous studies that demonstrated a higher risk of corneal graft rejection in young recipients [8,14,23–25]. This phenomenon has been observed in other solid organ transplantations and could be explained by a more intense immune system found during adolescence and in young adults compared to those with advanced age [26].

Regraft is considered as one risk factor for graft rejection and failure [27]. This might be caused from presensitization, immune memory, and inflamed and vascular surroundings occurred after previous PK [3]. The Collaborative Corneal Transplantation Studies (CCTS) research group showed an increase risk of graft rejection in regraft, especially when 2 or more grafts failed [8]. We also found that patients with multiple PK (> 2 times) had 2.7 times higher risk for rejection-related graft failure compared to patients who underwent PK 2 times. We found that approximately 40% of our patients developed corneal graft rejection. This finding differed from a previous study which was conducted in repeat PK patients [28]. The authors showed that only 5.2% of patients in the series developed graft rejection after surgery and a higher frequency of regraft was not significantly associated with graft failure [28]. This discrepancy of the results could be caused from a difference in patient characteristics and immunosuppressives used during operative period. The previous study used an intensive systemic steroid during the operative period (125 mg of methylprednisolone at immediately before surgery and 4 mg of betamethasone for 2 days postoperatively followed by 1 mg of betamethasone for 5 days) to minimize allograft rejection [28]. Unfortunately, only 7 (10.1%) of the 69 patients in our study received systemic immunosuppressive drugs (5 patients with cyclosporine A and 2 patients with mycophenolate mofetil) postoperatively.

This study did have some limitations due to the nature of the retrospective study design. The effect of HLA matching or ABO compatibility between donor and recipient on graft rejection in patients with repeat PK could not be evaluated and adjusted in the analysis because corneal tissue matching was not included in the criteria for donor allocation of the national eye bank in our country. Our study has a small sample size due to the strict inclusion criteria of regraft. However, to the best of our knowledge, this is the only study focusing on the effect of gender mismatch in patients with repeat PK and demonstrated no significant association between gender mismatch and poor survival outcome of repeat PK. We also performed a subgroup analysis in patients with available data of the first graft and found no significant association between gender mismatch of the first graft and graft rejection or rejection-related graft failure in repeat grafts. Therefore, the effect of gender mismatch of the first graft is unlikely to be masked by other risks associated with repeat PK.

## Conclusion

In summary, we did not observe the effect of gender mismatch on corneal graft rejection and rejection-related graft failure in patients with repeat PK. Young recipients and multiple PK were significantly associated with higher risk of graft rejection and rejection-related graft failure, respectively. Our results support previous evidence [17,18] that found that gender matching might not be compulsory for PK including patients with history of graft failure. Further prospective studies with a larger sample size are required to establish the benefit of gender matching between donor and recipient for high-risk corneal transplantation including those patients with repeat PK.

## Supporting information

S1 File (XLS)
